# Individual and combined effects of low dissolved oxygen and low pH on survival of early stage larval blue crabs, *Callinectes sapidus*

**DOI:** 10.1371/journal.pone.0208629

**Published:** 2018-12-07

**Authors:** Stephen J. Tomasetti, Brooke K. Morrell, Lucas R. Merlo, Christopher J. Gobler

**Affiliations:** School of Marine and Atmospheric Sciences, Stony Brook University, Southampton, New York, United States of America; University of Hong Kong, HONG KONG

## Abstract

A large number of coastal ecosystems globally are subjected to concurrent hypoxic and acidified conditions that will likely intensify and expand with continued climate change. In temperate regions, the spawning of many important organisms including the Atlantic blue crab *Callinectes sapidus* occurs during the summer months when the severity of coastal hypoxia and acidification is the greatest. While the blue crab earliest larval stage can be exposed to co-occurring hypoxia and acidification observed in many coastal ecosystems, the effects of these concurrent stressors on larval blue crab survival is unknown. This study investigated the individual and combined consequences of low dissolved oxygen (DO) and low pH on blue crab larvae survival through a series of short-term experiments. During 14-day experiments with moderately hypoxic conditions (117–127 μM O_2_ or 3.74–4.06 mg L^-1^) and acidified conditions (pH on total scale of 7.16–7.33), low DO and low pH individually and significantly reduced larval survival by 60% and 49%, respectively, with the combination of stressors reducing survival by 87% compared to the control treatment (210–269 μM O_2_ or 6.72–8.61 mg L^-1^, 7.91–7.94 DO and pH, respectively). During 4-day experiments with lower DO levels (68–83 μM O_2_ or 2.18–2.62 mg L^-1^) and comparable pH levels of 7.29–7.39, low DO individually reduced survival by >90% compared to the control (261–267 μM O_2_ or 8.35–8.54 mg L^-1^, 7.92–7.97 DO and pH, respectively), whereas low pH had no effect and there was no interaction between stressors. Over a 4-day period, the DO threshold at which 50% of the larval blue crab population died (LC_50_) was 121 μM O_2_ (3.86 mgL^-1^). In 14-day experiments, the DO and pH effects were additive, yielding survival rates lower than the individual treatments, and significantly correlated with DO and pH concentrations. Collectively, these findings indicate that blue crab sensitivity to both low DO and low pH are acute within the larval stage, depend on the intensity and duration of exposure, and leads to mortality, thereby potentially contributing to the interannual variability and possible regional declines of this fishery.

## Introduction

Hypoxic waters are expanding globally and are a ubiquitous characteristic of coastal ecosystems along the Atlantic and Gulf coasts of the United States [1, 2]. The primary driver of coastal hypoxia is eutrophication [3–5]. Nutrient enrichment stimulates phytoplankton primary production, and excessive algal biomass is consumed through microbial aerobic respiration, depleting oxygen in the water. In addition to the reduction of dissolved oxygen (DO) another consequence of microbial respiration is the production of CO_2_, and subsequent lowering of seawater pH [6–9].

The metabolically mediated co-occurrence of acidification and hypoxia in coastal environments has been well established over a range of spatial and temporal scales [6, 10]. Coupled fluctuations of DO and pH linked to seasonal, diurnal, and tidal trends, can expose organisms to acidified and hypoxic conditions, even in shallow, nearshore habitats where stratification is rare [10, 11]. Some coastal ecosystems already experience pH values lower than those predicted to occur in the open ocean by 2100 [12], and the acidification of these systems will be intensified by the climate change-associated acidification of the future [13].

Independently, the biological implications of hypoxia and acidification are well studied. Lethal and sublethal oxygen minimum thresholds vary among taxa and can exceed 2.0 mg L^-1^, a common regulatory standard [14]. Organisms able to withstand periods of hypoxia may experience sublethal stress in the form of reduced growth, feeding, locomotion, or fecundity [15, 16]. Furthermore, altered physiology or behavior in response to lowered DO can make some organisms more vulnerable to predation [16] as well as other co-stressors [17, 18]. Surveys of the biological implications of ocean acidification have primarily focused on calcifying marine organisms [19–21] because of their physiological dependence on the availability of CO_3_^2-^ ions. Results of a meta-analysis among calcifying organisms indicate negative effects on reproduction, growth, calcification, and survival that vary across taxa and life stage [22].

Among calcifying crustaceans, some individual species examined to date have exhibited steady or increased calcification under acidified conditions [21, 23]. However, increased calcification could have associated costs, such as the decreased growth observed in juvenile Red king crabs *Paralithodes camtschaticus* [24]. Other crustaceans such as Tanner crab *Chionoecetes bairdi*, have exhibited decreased calcification in response to acidification [24]. Acidification could also affect benthic decapod survival at various life stages. Juvenile *C*. *bairdi* and *P*. *camtschaticus* both exhibited increased mortality when exposed for 200 days to waters at or below saturation with regard to calcite [24]. Moreover, exposure of *P*. *camtschaticus* to low pH conditions increased mortality at both embryonic and larval phases and had an additive effect between phases [25].

Despite the coupled nature of hypoxia and acidification in coastal ecosystems, the effects of low DO and low pH on economically and ecologically important organisms are largely unknown [8]. Seasonal hypoxia and simultaneous acidified conditions can occur at the same time as spawning events of important fish, mollusks, and crustaceans, potentially affecting their larvae. Early life stages of calcifying bivalves such as hard clams (*Mercenaria mercenaria*) and bay scallops (*Argopecten irradians*) exhibited additive and synergistic negative responses to hypoxia and acidification that include slower growth rates, impaired survival, and an inability to metamorphose [18]. Additionally, the larval stage of *Menidia* spp., ecologically important forage fish, displayed dramatic declines in post-hatch survival when exposed to both low pH and low DO [26]. The effects of co-occurring low DO and low pH on the larval stages of other commercial and ecologically important coastal marine organisms are scarce, representing a knowledge gap in the literature.

The blue crab, *Callinectes sapidus*, is a key fishery along the Gulf and Atlantic coasts of the United States with landings having exceeded 60 metric tons, and more than $175M, annually from 2010–2016 (National Marine Fisheries Service, Fisheries Statistics Division, 2017). Blue crabs also play a key role within estuarine ecosystems [27] as keystone predators [28], recyclers of carbon from the benthos to the nekton and vice versa [29], and potential biotic resistance to invasive species such as the introduced green crab, *Carcinus maenas* [30]. The life cycle of *C*. *sapidus*, involves a combination of estuarine and marine habitats. Mating occurs in low-salinity estuary waters following the females’ terminal molt [31], the timing of which varies regionally [32]. After copulation, females migrate toward higher salinity waters at the mouth of estuaries to spawn [33]. Newly hatched blue crab larvae can spend up to the first two zoeal (larval) stages in estuarine or near-shore habitats before being transported to continental shelf waters by seaward currents thereafter [34–37]. Although early stage zoea may be exposed to low DO and pH conditions typical of estuarine ecosystems, there is little understanding with regard to the concurrent effects of these stressors on the survival of blue crab larvae.

Some of the earliest work investigating the impacts of hypoxia and elevated CO_2_ involved adult blue crabs [7]. Adult blue crabs are generally considered tolerant of hypoxia and acidification because of their ability to regulate HCO_3_^-^ [7], and increase high oxygen-affinity respiratory pigments through a hypoxia-stimulated structural change in their blood [38]. Despite their ability to regulate hemolymph chemistry, exposure to low pH and low DO can impair the immune response, slowing the rate of clearance of bacteria from the hemolymph [39]. Additionally, low DO and low pH conditions can significantly reduce the activity (up to 70%) of the terminal enzyme of the prophenoloxidase activating system, a major defense system of the blue crab [40]. Despite this refined understanding established by previous studies of the effects of the combination of low DO and low pH on adult blue crabs, there are no published studies investigating the effects of these concurrent stressors on blue crab larvae. However, in temperate regions significant overlap occurs between spawning season and the occurrence of concurrent hypoxia and acidification [6, 11, 41, 42]. In terms of acidification, a recent study with larval blue crabs revealed a 23% reduction in survival of newly hatched *C*. *sapidus* zoea when exposed to pH of 7.8 compared to the control pH of 8.2 [43]. Moreover, 38% of the OA experimental replicates experienced complete population crashes (not counted in mortality data), in which the population was suddenly reduced to <1% of its original size, whereas there were no such population crashes in the control treatments [43]. While the pH levels utilized by Giltz and Taylor [43] were designated to simulate current and future pH of the open ocean, some blue crab stage one zoea are likely to be exposed to both reduced pH and reduced DO levels typical of estuarine and coastal systems [6, 13] and thus lower than used in this prior study.

Here, we examined the effects of low DO and low pH, individually and in combination, on the survival of early zoeal stages of *C*. *sapidus*. A series of laboratory experiments were conducted using mixtures of air and tanked gases of CO_2_ and N_2_ to manipulate DO, pCO_2_ and pH in an effort to test our hypothesis that the concurrent stressors will negatively affect zoeal survival. Multiple experiments were run to account for differing cohorts of larvae, using differing durations and intensity of exposure to low oxygen and low pH conditions to allow for a refined sense of the specific sensitivity of *C*. *sapidus* larvae.

## Methods

This study presents five experiments examining the individual and combined effects of low DO and low pH on *C*. *sapidus* larvae. Each experiment involved newly hatched zoea under two oxygen conditions and two pH levels, and persisted either four or fourteen days. Daily measurements of temperature, salinity, DO, and pH were recorded for experiments, and larval survival was assessed approximately every three days in 14-day experiments and at the end of all 4-day experiments. Salinity and temperature were maintained at 30±1 and 25±1°C respectively, levels appropriate for the development of *C*. *sapidus* larvae in the laboratory [44]. Experiments differed with regard to sources of larvae, duration, target DO and pH levels, and starting zoeal density per vessel (Table 1). Experiments differed in sources of larvae to determine if regional and maternal effects may drive experimental outcomes, while differences in duration, target DO, and target pH levels between experiments were designed to identify potential DO and/or pH mortality thresholds. All larval densities were within the range shown to produce maximal survival [45]. Target DO and pH levels were chosen based on published DO and pH values in eutrophic coastal zones [6, 10, 13]. Experiments persisting 14 days were chosen to simulate the development time from larval hatch to the second molt [44], while 4-day experiments simulated shorter retention times in estuaries. All experiments were performed at Stony Brook University’s Marine Science Center located in Southampton, NY, USA.

**Table 1:** Source of larvae, duration, target low pH_T_ (pH_T_ = pH in total scale), target low dissolved oxygen (DO), and initial larval density during experiments in which larval stage *Callinectes sapidus* were exposed to differing levels of pH and DO achieved via mixing tanked gases.

Experiment	Source of larvae	Duration (d)	Target low pH_T_	Target low oxygen (μM O_2_) / (mgL^-1^)	Initial larval density
1	Southampton, NY	14	7.3	120 / 3.84	71 L^-1^
*2	Southampton, NY	14	7.2	120 / 3.84	100 L^-1^
3	Baltimore, MD	14	7.2	120 / 3.84	50 L^-1^
4	Baltimore, MD	4	7.3	70 / 2.24	125–75 L^-1^
5	Baltimore, MD	4	7.3	80 / 2.56	100 L^-1^

* larvae from 4 females; all chemistry parameters are presented in S1 and S2 Tables.

### Experimental design

Experiments were developed to follow the factorial low oxygen x low pH experimental approach outlined by Gobler and Baumann [8]. Heating wands (Finnex Aquarium Heaters) maintained water temperatures of 25±1°C that circulated through a sea table via multiple airlines. Sixteen 1-L polyethylene vessels containing 0.2μm filtered seawater from eastern Shinnecock Bay, NY, USA, were placed within a sea table and covered with polycarbonate lids. Replicate (n = 4) vessels received mixtures of CO_2_ gas, N_2_ gas, and air [18] at different rates enabling the maintenance of normoxic and normal pH conditions (control treatment), normoxic and low pH conditions (low pH treatment), hypoxic and normal pH conditions (low oxygen treatment), and hypoxic and low pH conditions (low oxygen-low pH treatment). Gas proportioners (Cole-Parmer Flowmeter System, multitube frame) were used to deliver mixtures of gases continuously through plastic airlines (Tygon) connected to glass-bonded silica air stones placed within the bottom of each vessel. The rate of gas delivery (~300 mL min^-1^) allowed for complete turnover of the vessel volumes many times hourly, maintaining consistent conditions within each vessel. The control treatment vessels were aerated with ambient air. The low oxygen treatment vessels were bubbled with 400 parts per million (ppm) CO_2_/N_2_ mixed gas and ambient air [18]. The low pH treatment vessels were bubbled with 5% CO_2_ gas and ambient air. The low oxygen-low pH treatment vessels were bubbled with 5% CO_2_, N_2_ gas, and ambient air [18].

### Blue crab larvae

Ovigerous females were collected in Shinnecock Bay, NY, USA, prior to the start of some experiments during summer July-September months. Permission to collect blue crabs from Shinnecock Bay was granted by the New York State Department of Environmental Conservation via a Scientific Collectors Permit. Females were placed in 20-L polyethylene buckets aerated with ambient air via plastic airlines attached to glass bonded silica air stones, or partitioned by mesh partitions within a sea table, until hatching. Upon hatching, zoea were concentrated in a 10-L polyethylene vessel, and distributed into each experimental vessel according to densities listed in Table 1. Densities never exceeded 125 larvae L^-1^ to maximize survival of larvae [45]. Daily, larvae were fed live rotifers (50 individuals mL^-1^) enriched with *Nannochloropsis* [45]. After ~10 days zoea were additionally fed live *Artemia* nauplii, at densities of 300 individuals L^-1^ [45]. In some cases when ovigerous females were not available, newly hatched larvae were shipped overnight to the Stony Brook Marine Station from the Institute of Marine and Environmental Technology, Department of Marine Biotechnology of the University of Maryland, Baltimore County, and experiments proceeded as previously described. Ovigerous females from the University of Maryland were obtained from crabbers at the end of summer, quarantined in 15°C tanks, and then moved to 22°C tanks to prepare for spawning. Both larvae shipped to Stony Brook Marine Station, Southampton NY, and larvae from Shinnecock Bay broodstock were from single mothers, except in one case, when larvae from four females from Shinnecock Bay hatched simultaneously (Table 1).

Approximately every three days, survival was quantified and vessel seawater was exchanged. Zoea were carefully poured onto a 250μm sieve, and transferred into a small glass dish of filtered seawater where live individuals were quantified. Counts were made three times per replicate and averaged for each time point. Individuals were considered dead if they had undergone decay and did not respond to light. Dead individuals were removed when densities were low enough to allow for removal without pipetting live individuals. All vessels were rinsed with 1% Alconox detergent solution, liberally rinsed with fresh water, and rinsed and filled with new filtered seawater before returning individuals to vessels. Zoea were fed within an hour of completion of the water change.

### Water chemistry

Measurements of temperature, DO, and pH were made daily. Temperature was measured with a digital thermometer. An YSI 5100 oxygen meter with a Clark-type electrode, calibrated daily, was used to measure DO. This probe yields values that are not significantly different than values obtained from Winkler titrations [18]. A Honeywell Durafet Ion Sensitive Field Effect Transistor (ISFET)-based pH sensor calibrated with Dr. Andrew Dickson’s (University of California San Diego, Scripps Institution of Oceanography) seawater pH standard [46], was used to make discrete pH readings (total scale). This pH sensor provides measurements consistent with spectrophotometric measurements of pH using m-cresol purple [47].

Dissolved inorganic carbon (DIC) analyses were performed at the beginning and/or end of experiments to characterize the carbonate chemistry of each treatment throughout experiments. A VINDTA 3D system was used to extract DIC from experimental samples and transfer released CO_2_ to a UIC 5011 coulometric titration unit to measure DIC. Dr. Andrew Dickson’s certified reference material (Batch 117 = 2009.99 μmol DIC kg seawater^-1^ and Batch 158 = 2043.54 μmol DIC kg seawater^-1^) was analyzed before each set of analyses, which only proceeded when recovery of CRM exceeded 99.6%. The CO_2_SYS program (http://cdiac.ornl.gov/ftp/co2sys/) was used to calculate levels of CO_2_ and the complete carbonate chemistry of seawater, using measured values of DIC, pH, temperature, salinity, pressure, phosphate, silicate, and H_2_CO_3_ dissociation constants [48].

### Statistical analysis

Two-Way Analysis of Variance (ANOVA) tests were performed to assess differences in survival data between treatments. Percent survival values were arc-sin square root transformed before analysis. ANOVA assumptions were assessed via Shapiro-Wilk tests of normality and Bartlett’s tests for homogeneity of variance. Two-Way ANOVAs were followed by Tukey’s multiple comparison tests. ANOVA tests or Kruskal-Wallis tests were used to assess differences in pH and DO among treatments in the various experiments. Statistical analyses were completed with RStudio statistical software (Version 1.0.153). Linear and logistic regressions for meta-analysis of experiments were performed using Sigmaplot statistical software (Version 11, Systat Software, Inc.). Three-parameter logistic regressions were performed on DO-percent survival data, and pH-percent survival data to determine potential DO and pH thresholds for zoeal survival. If relationships were not significant, linear regressions were performed. Survival values are reported as means ± standard error. Seawater chemistry values are reported as means ± standard deviations, with temperature, DO and pH values derived from daily measurements of these parameters in all replicates per treatment, and all other measured and calculated parameters derived from DIC measurements among the replicates of each treatment from the start and/or end of experiments.

## Results

In all five experiments (three 14-day and two 4-day), low DO and low pH treatments had DO and pH levels significantly lower than control treatment DO and pH levels (p<0.05, ANOVA or Kruskal-Wallis Test; S1 and S2 Tables).

### Fourteen-day experiments

**Fig 1:**
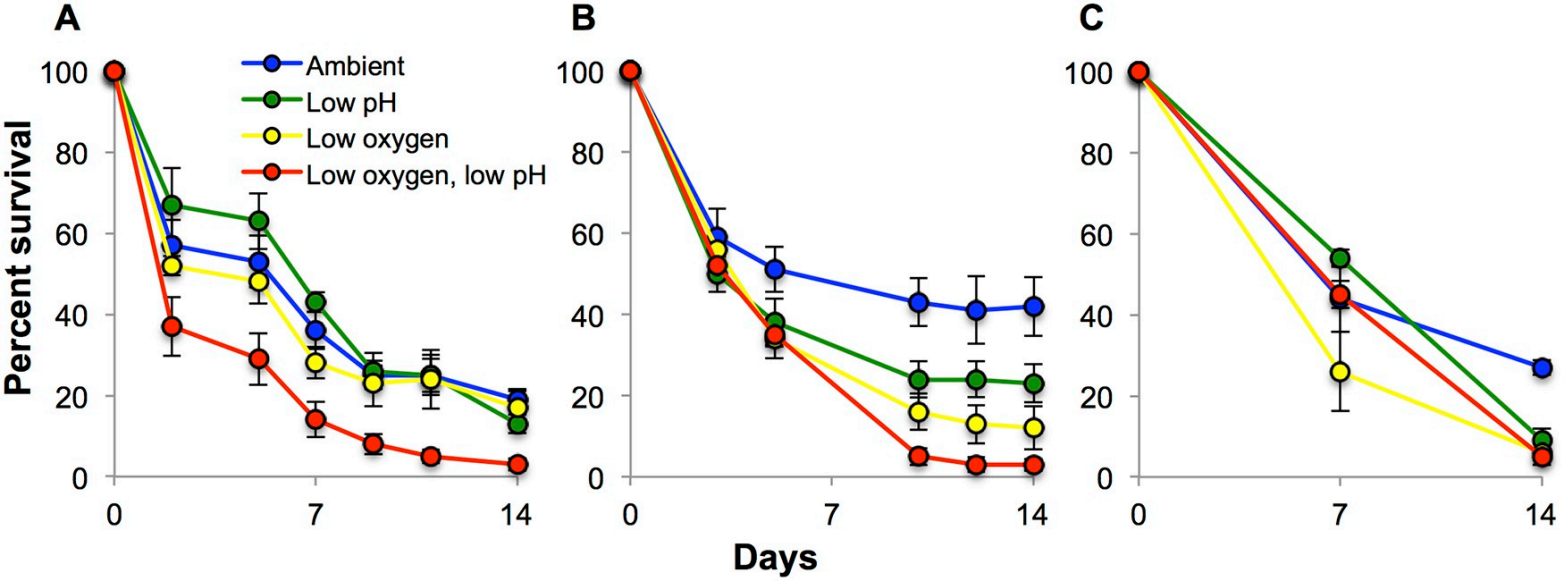
Percent survival of *C*. *sapidus* larvae exposed to two oxygen and pH levels over fourteen days. (A) Percent survival of *C*. *sapidus* larvae during the first 14-day experiment, (B) second 14-day experiment, and (C) third 14-day experiment. Each experiment was performed with a different cohort of larvae, with the second experimental cohort consisting of multiple mothers. The third experiment contained fewer time points due to logistical constraints. Points are means ± standard error. Seawater chemistry is presented in S1 Table. Statistical analysis is presented in S3 Table.

In all three 14-day experiments where *C*. *sapidus* larvae were exposed to two levels of DO and two pH levels, low DO and low pH significantly reduced zoeal survival (*p*<0.05; Fig 1; S3 Table), and their combination elicited additive effects. In the first 14-day experiment, exposure to low DO (120±7.42 μM O_2_) and exposure to low pH (7.32±0.06) both individually and significantly reduced larval survival from 19% after 14 days (control) to 17% and 13% respectively (p<0.05 and p<0.005 respectively, Two-Way ANOVA), with their combination having an additive effect (3% survival, Fig 1A; Two-Way ANOVA; S3 Table).

At the completion of the second 14-day experiment, a significant reduction in zoeal survival occurred for the low pH treatment (7.20±0.06; p<0.05) and low oxygen treatment (121±18.4 μM O_2_; p<0.001, Two-Way ANOVA; S3 Table), with survival measured at 42%, 23%, 12%, and 3% for control, low pH, low oxygen, and combined low oxygen-low pH treatments, respectively (Fig 1B), with no interaction detected. In the third 14-day experiment, both low oxygen (126±15.9 μM O_2_) and low pH (7.27±0.08) had significant negative effects (p<0.005, p<0.01, respectively, Two-Way ANOVA) on zoeal survival, reducing survival from 27% in the control to 6% and 9% low oxygen and low pH, respectively, with the combined effect being additive and yielding 5% zoeal survival (Fig 1C; S3 Table).

### Four-day experiments

**Fig 2:**
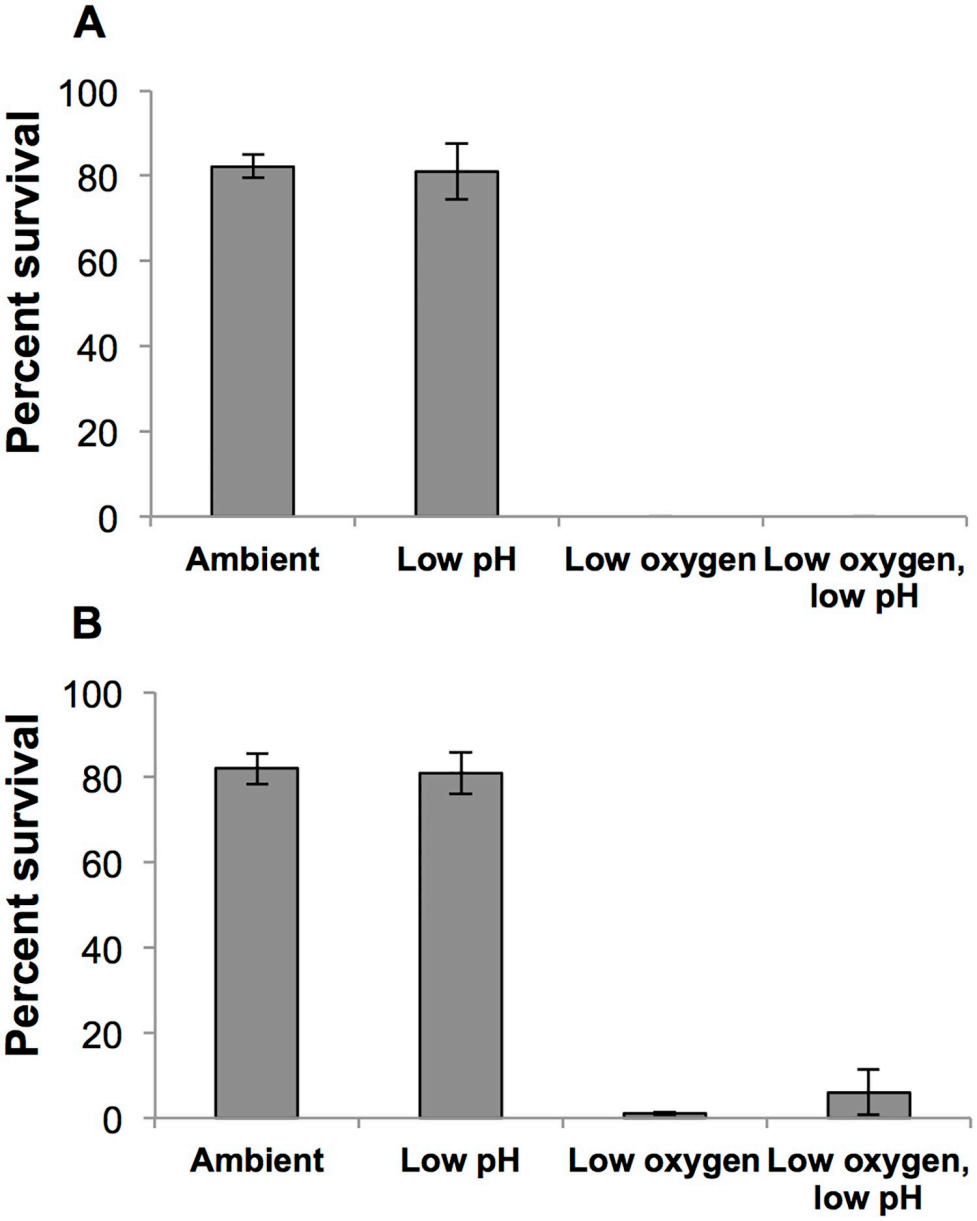
Percent survival of *C*. *sapidus* larvae exposed to two oxygen and pH levels over four days. (A) Percent survival of *C*. *sapidus* larvae during the first 4-day experiment, and (B) second 4-day experiment. Bars are means ± standard error. Seawater chemistry is presented in S2 Table. Statistical analysis is presented in S4 Table.

In both 4-day experiments where *C*. *sapidus* larvae were exposed to two levels of DO and two pH levels, low DO significantly reduced zoeal survival (p<0.001, 2-way ANOVAs), low pH had no effect, and no interaction was detected (Fig 2; S4 Table). In the first of the two 4-day experiments zoea from the low oxygen and combined low oxygen-low pH treatments (72.4±16.9 μM O_2_) experienced complete mortality after four days of exposure to low oxygen values (p<0.001, Two-Way ANOVA; Fig 2A; S4 Table). Zoea were resistant to low pH (7.39±0.05) at normal oxygen levels, and experienced 81% survival which was not significantly different from the control treatment survival of 82% (Fig 2A; S4 Table).

In the second 4-day experiment, DO values were slightly higher than the first 4-day experiment to increase survivorship (S2 Table). Larval blue crabs experienced significantly reduced survival in response to low DO (80.2±7.37 μM O_2_; p<0.001 Two-Way ANOVA), but not low pH (7.34±0.06), and there was no interaction between the treatments (Fig 2B; S4 Table). Total survivorship for control, low pH, low oxygen, and combined low oxygen low pH over four days were 82%, 81%, 1%, and 6% (Fig 2B).

### Meta-analyses of experiments

**Fig 3:**
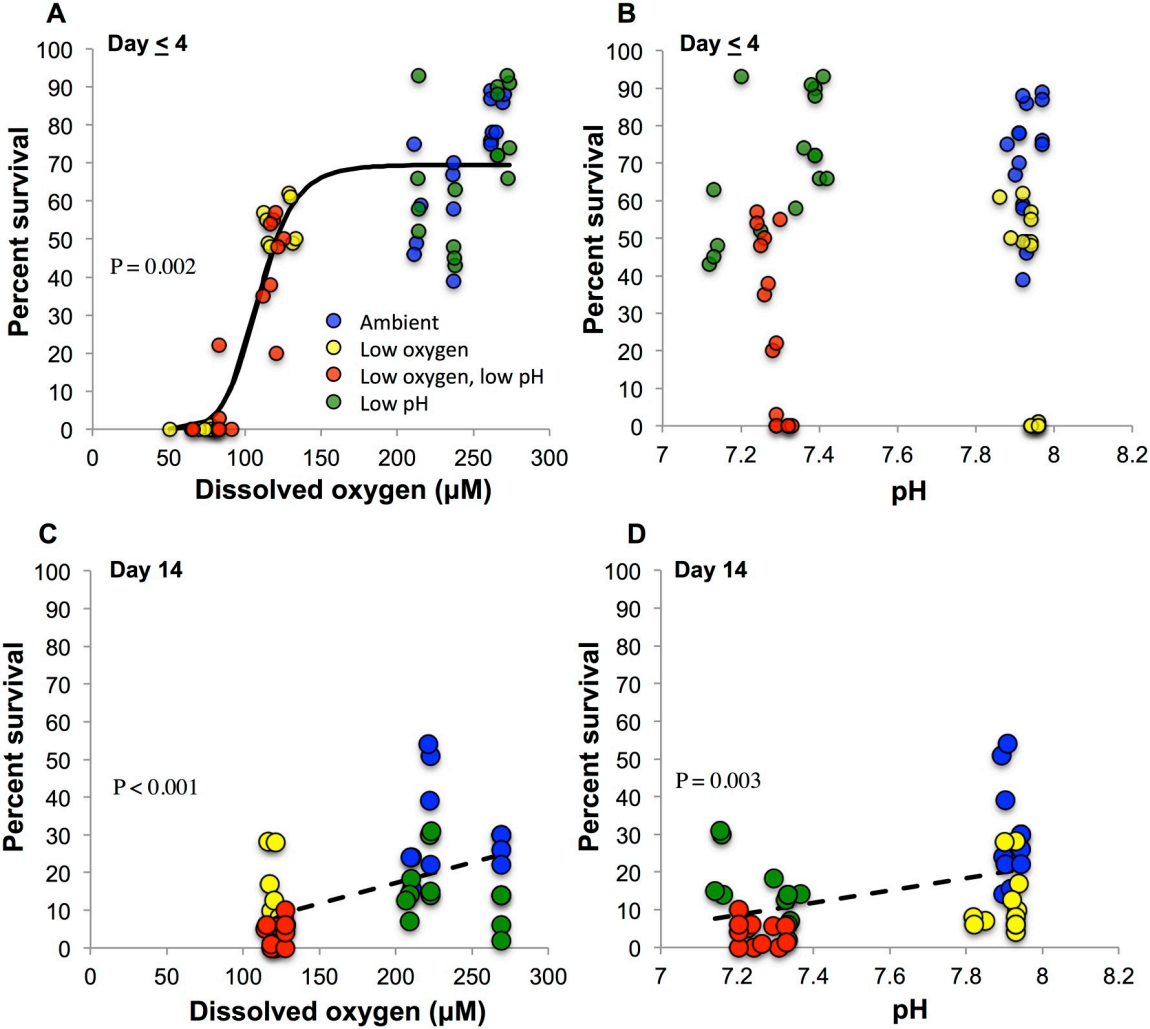
Logistic and linear regressions of experimental replicates. (A) Percent survival of *C*. *sapidus* larvae plotted with DO from the first time point of each replicate of each experiment excluding experiment 3 which did not have a 4-day time point. Statistical analysis is presented in S5 Table. (B) Percent survival of *C*. *sapidus* larvae of plotted with pH from the first time point of each replicate of each experiment excluding experiment 3 which did not have a 4-day time point. (C) Percent survival of *C*. *sapidus* larvae of plotted with DO of each replicate of each 14-day experiment. Statistical analysis is presented in S6 Table. (D) Percent survival of *C*. *sapidus* larvae of plotted with pH of each replicate of each 14-day experiment. Statistical analysis is presented in S7 Table.

The percent survival data from the first time point of all replicates of four of five experiments were plotted as a function of both DO and pH (Fig 3A and 3B). The data from the third 14-day experiment was excluded because the first time point at which percent survival was quantified occurred 7 days into the experiment, 3–5 days later than the other experiments. A 3-parameter logistic curve was fitted to the DO—percent survival data that yielded an R^2^ value of 0.82 (Fig 3A; S5 Table). Over less than four days of exposure, the DO threshold at which 50% of the larval blue crab population died (LC_50_) was 120.6 μM O_2_ or 3.86 mgL^-1^ (Fig 3A). There was not a significant logistic or linear relationship between pH levels used and survival at the first time point of 4-day experiments (Fig 3B). No significant logistic relationships were detected between larval survival and DO or pH at 14-days of exposure. Linear regression analyses, however, indicated a statistically significant correlation between percent larval survival and DO concentration (R^2^ = 0.26, p<0.001, S6 Table; Fig 3C) and between percent larval survival and pH (R^2^ = 0.18, p<0.01, S7 Table; Fig 3D) after 14 days of exposure.

## Discussion

In this study, early stage larval blue crabs were exposed to two levels of DO and pH for either four or fourteen days. Low DO had the greatest effect on zoeal survival at both four and fourteen days, while low pH only had an effect after the longer exposure. In all 14-day experiments with DO at sublethal levels, the combination of low DO and low pH produced additively negative effects on zoeal survival.

Our findings are consistent with prior studies that have documented increases in larval crab mortality in response to reductions in DO [49]. Crustaceans are among the most sensitive taxa to low DO [14], with pelagic larvae being the most vulnerable life-stage [49]. Crustacean larvae may be most sensitive to hypoxia during the first molt-cycle and have been shown to be more sensitive at day four than earlier days, exhibiting mortality at higher DO levels when exposure persisted for four days compared to lower DO levels at one day of exposure [49]. During the current study, over a period ≤ four days, the DO concentration at which 50% of the blue crab larvae died (LC_50_) was 120.6 μM O_2_ (Fig 3A), or 3.86 mgL^-1^. This threshold is higher than seven other larval crustacean species tested in a study containing similar 4-day experiments, where the average LC_50_ value for the tested species was 2.31 mgL^-1^ [49]. Together these results suggest that blue crab larvae are among the most sensitive crustacean larvae to hypoxia, and even slight reductions in DO concentrations may increase mortality rates and potentially increase their vulnerability to other environmental stressors. Additionally, the presence of other stressors may further reduce the narrow physiological range of optimal DO concentrations [50, 51].

Regarding acidification, blue crabs exhibit a variety of responses to lowered pH depending on life-stage [21, 52–54] with observed negative effects such as reductions in growth or survival mainly occurring in the larval stages [43]. Our study found significant reductions in zoeal survival after fourteen, but not four, days of exposure to low pH conditions ranging from 7.2–7.32 (Fig 1). Since our low pH levels were chosen to represent eutrophic, coastal systems [6, 13] they were lower than those used by Giltz and Taylor [43]. Our results were consistent with Giltz and Taylor [43], however, who found that acidification reduced zoeal survival over a period exceeding 10 days. Our study incorporated both low DO and low pH, and found that low pH can act as a secondary stressor, producing additively negative effects on zoeal survival. Conversely, larval blue crab survival was unaffected by low pH conditions in 4-day experiments at similar pH levels (Fig 2; S1 and S2 Tables), suggesting that exposure must be prolonged to affect survival. In all experiments, treatments containing low pH were undersaturated in calcite (with average Ω_calcite_ of 0.55–0.97) while the other treatments were supersaturated (average Ω_calcite_ of 2.79–3.48, S1 and S2 Tables). The carapace of decapod crustaceans such as the blue crabs is comprised of amorphous calcium carbonate containing high amounts of magnesium packed within a chitin-protein matrix [53, 55, 56], and it may be that calcite undersaturation affects blue crab larvae calcification impacting survival. Molt-related mortality may also be the result of challenges in the regulation of acid-base balance, as suggested by Small et al. [57] explanation of increased mortality among juvenile European lobsters exposed to elevated pCO_2_. For blue crab larvae, the energetic strains of acid-base regulation, in conjunction with increased molting frequency of larval stages may result in reduced tolerance of acidified conditions. More information regarding the acid-base regulatory adaptations of early life stage invertebrates and the resulting sensitivities to high CO_2_ is needed [58]. Although, no specific pH thresholds were detected during this study, 14-day survival significantly correlated with pH levels (Fig 3D) suggesting that, over longer timescales, low pH may impact calcification and/or energy allocations. Further efforts are needed clarify the range of concentrations and time scales at which low pH has deleterious effects on larval blue crab survival.

An increasing emphasis on multiple stressors has led to the development of bioenergetic frameworks for understanding the individual and combined effects of stressors on specific species and life-stages and predicting future ecosystem-level change [50, 51]. Concurrent stressors such as hypoxia and acidification can decrease an organism’s aerobic scope (or fraction of energy available beyond the basal metabolic requirements) and can sometimes interact in a synergistic manner [50]. Extreme stress can induce coping strategies such as metabolic depression [17] and shifts to anaerobic pathways [50] although these defenses are limited in their capacity under intensifying physiological stress [17] and the inability to meet the energy demand for basal metabolic needs can eventually lead to a negative aerobic scope and death [50]. Curiously, crustaceans are often regarded as tolerant of ocean acidification [22, 59] and sensitive to hypoxia [14, 60], so they may serve as a unique model taxa to further explore the combined effects of low DO and low pH [60], especially considering the range of conditions crustacean larvae may be exposed to throughout prolonged periods of larval development during dispersal in coastal ecosystems.

Our findings contrast with the only prior hypoxia-acidification study involving decapod crustaceans which involved adults and saw no significant mortality as a result of low DO and low pH [61]. While the available comparisons are limited, this suggests that the combined effects of hypoxia and acidification on decapod crustaceans are life-stage dependent, consistent with prior studies on hypoxia [49] and acidification [25, 43]. The experimental outcomes with larvae from Baltimore and from Southampton were highly similar, both demonstrating low DO effects and low pH effects on larval survival over 14 days (Fig 1). Similarly, experimental results with larvae supplied from single mothers were highly similar to the experimental results from larvae supplied from four mothers, suggesting maternal effects did not drive experimental outcomes (Table 1, Fig 1).

A study of thermal tolerances of Chilean kelp crab larvae, *Taliepus dentatus* may provide insight into the potential mechanisms for reduced survival of blue crab larvae exposed to low DO and low pH [62]. In that study, stress associated with warming above a threshold temperature reduced aerobic scope and the capacity to provide oxygen to the tissues could not meet the demand [62]. Similarly in our study, reduced concentrations of DO may have limited the supply of oxygen to the tissues; low pH exposure for long enough duration may further limit aerobic scope by providing an energetic strain on blue crab larvae through the induction of energy intensive acid-base regulatory pathways [23, 63]. Thus, low DO and low pH in tandem might reduce aerobic scope to negative values and result in enhanced larval mortality.

Concurrent hypoxia and acidification, along with ocean warming, typify the environmental stressors of marine climate change [12, 51]. Future projections indicate nonlinear increases of *p*CO_2_ in coastal hypoxic zones [13, 64], and intensification of coastal hypoxia in some eutrophic systems owing to the influence of warming on respiration, precipitation, stratification and oxygen solubility [2, 65]. However, individual coastal environments may exhibit differential responses because of diverse morphology and circulation, regional climate, and anthropogenic impacts across systems. Additionally, these factors can also impact the pre-existing baseline variability in respective systems [11], which are important considerations for multifactorial experiments designed to understand the biological implications of environmental stressors. Dynamic coastal environments will expose organisms to naturally fluctuating intensities of stress that can vary by life-stage [66]. Hypoxia-acidification multifactorial experiments to date have been performed using both chronic [18, 26, 67] and diurnally fluctuating DO and pH [68–70]. While stratified estuaries can experience extended periods of chronic hypoxia and acidification (i.e. weeks) [6] coastal lagoons such as Shinnecock Bay, NY, USA, where some of the ovigerous females used as broodstock were found, experience large diurnal fluctuations in DO, CO_2_ and pH, with daytime photosynthesis alleviating low DO, low pH conditions [7, 10]. Hypoxia, acidification, and diurnal fluctuations of these parameters can also vary seasonally, with the greatest intensity, largest fluctuations, and lowest daily averages occurring during summer and early fall months [6, 11]. Given the rapid and mass mortality before the first time point in the hypoxia treatments of our 4-day experiments, it would seem cohorts of blue crabs spawned in eutrophic estuaries during this late summer and early fall when hypoxia is most intense would be more likely to perish than those spawned earlier in the year. Furthermore, some studies suggests that synchronized hatches occur at night-time on ebb tide [71–73], when both DO and pH may be minimal [10].

Behavioral adaptations of the blue crab larvae may limit their exposure to low DO and low pH conditions in estuaries. Upon hatching, stage I zoea exhibit negative geotaxis, swimming at rates of 0.5 cm s^-1^ toward the surface waters [74], where DO and pH values are likely higher than at depth [6]. Moreover, the surface-dwelling larvae are transported out of estuaries by seaward surface flow [73]. Although stage II zoea are found in estuarine and nearshore habitats, stage I zoea strongly outnumber the other stages sampled [34, 35]. Thus, it is likely that only a small fraction of blue crab larvae may be exposed to two full weeks of low DO and low pH conditions. Stage I zoea, which are most often found in estuaries, molt to stage II sometime between 5–13 days depending on temperature and salinity characteristics [44], which is within the temporal range of our experimental design. Nearly all crab larvae perished in low DO treatments after four days and thus it is likely that significant reductions in survival are experienced by stage I zoea encountering such conditions for even shorter durations of time. Still, more investigation is needed into the effects of co-occurring hypoxia and acidification on blue crab larvae over multi-hour to multi-day timescales. Future short-term studies could also incorporate diurnal fluctuations of DO and pH into experiments with blue crab larvae to contrast those impacts with the chronic exposures utilized here.

The meroplanktonic life cycle and extended dispersal distances expose blue crab larvae to a range of environmental conditions with some earliest stage larvae likely experiencing concurrent low DO and low pH typical of net heterotrophic coastal environments. Long dispersal distances and recruitment into habitats that differ from the parental habitat may limit the populations’ ability to adapt to low DO and low pH, as a result of uneven selective pressures across habitats [75]. Hypoxia and acidification are generally expected to worsen with continued climate change [13, 65] and small changes in the rate of larval mortality can dramatically affect recruitment [75]. Embryonic stages may be exposed to longer, more severe durations of hypoxia and acidification, and the carryover effects of these stressors on the larval stages are unknown. The results of our study suggest that both hypoxia and acidification can affect blue crab larval survival independently and, when experienced concurrently, elicit an additive effect. Given that coastal hypoxia and acidification may contribute to blue crab population variability, future management plans of this important species that consider the influence of these stressors may improve the sustainability of this fishery.

## Supporting information

S1 Table Mean pH, carbonate chemistry, alkalinity, DO, temperature and salinity (± 1 SD) during experiments one, two, and three in which larval stage *Callinectes sapidus* were exposed to differing levels of pH and DO achieved via mixing tanked gases. (DOCX)

S2 Table Mean pH, carbonate chemistry, alkalinity, DO, temperature and salinity (± 1 SD) during experiments four and five in which larval stage *Callinectes sapidus* were exposed to differing levels of pH and DO achieved via mixing tanked gases. (DOCX)

S3 Table Experiments one, two, and three: Two-way analysis of variance for *Callinectes sapidus* larval survival when exposed to two levels of DO and pH for 14 days. (DOCX)

S4 Table Experiments four and five: Two-way analysis of variance for *Callinectes sapidus* larval survival when exposed to two levels of DO and pH for 4 days. (DOCX)

S5 Table Logistic, 3-parameter nonlinear regression for *Callinectes sapidus* larval survival when exposed to varying levels of DO for a period ≤ 4 days; Regression Equation: y = a / [1 +(x/x_0_)^b^]. (DOCX)

S6 Table Linear regression for *Callinectes sapidus* larval survival when exposed to varying DO levels for 14 days; Regression Equation: y = 0.109x – 4.554. (DOCX)

S7 Table Linear regression for *Callinectes sapidus* larval survival when exposed to varying pH levels for 14 days; Regression Equation: y = 16.325x – 108.96. (DOCX)

S1 File Larval survival data. (XLSX)
