# Supplementary material for: Individual and combined effects of low dissolved oxygen and low pH on survival of early stage larval blue crabs, Callinectes sapidus
Source: PLoS One. 2018 Dec 7;13(12):e0208629. doi: 10.1371/journal.pone.0208629 (PMC6285982; doi:10.1371/journal.pone.0208629)
Supplement: S1 Table — (DOCX) [file pone.0208629.s001.docx]

**S1 Table. Mean pH, carbonate chemistry, alkalinity, dissolved oxygen, temperature and salinity (± 1 SD) during experiments one, two, and three in which larval stage *Callinectes sapidus* were exposed to differing levels of pH and dissolved oxygen achieved via mixing tanked gases.** (DOC)

| **Experiment** | **Parameter** | **Control** | **Low oxygen** | **Low pH** | **Low oxygen, low pH** |
| --- | --- | --- | --- | --- | --- |
| Experiment 1 | pH_T_ | 7.91 ± 0.036 | 7.93 ± 0.015 | 7.33 ± 0.066 | 7.31 ± 0.046 |
|  | pCO_2_ (*μ*atm) | 600 ± 1.91 | 663 ± 44.2 | 3270 ± 475 | 3323 ± 12.7 |
|  | Ω_calcite_ | 2.89 ± 0.01 | 2.75 ± 0.26 | 0.68 ± 0.10 | 0.65 ± 0.003 |
|  | Total DIC (*μ*mol L^-1^) | 1805 ± 4.83 | 1839 ± 34.2 | 2008 ± 14.1 | 1984 ± 8.95 |
|  | CO_3_^2-^ (*μ*mol L^-1^) | 115 ± 0.255 | 109 ± 10.4 | 27.1 ± 3.81 | 25.7 ± 0.135 |
|  | Alkalinity (TA) | 1960 ± 5.01 | 1983 ± 49.7 | 1955 ± 5.21 | 1928 ± 8.73 |
|  | Salinity | 29.3 ± 1.0 | 29.3 ± 1.0 | 29.3 ± 1.0 | 29.3 ± 1.0 |
|  | Temperature (°C) | 25.5 ± 0.4 | 25.5 ± 0.4 | 25.5 ± 0.4 | 25.5 ± 0.4 |
|  | Dissolved oxygen (μM) | 210 ± 6.20 | 118 ± 5.61 | 209 ± 6.30 | 121 ± 8.65 |
| Experiment 2 | pH_T_ | 7.90 ± 0.019 | 7.85 ± 0.162 | 7.16 ± 0.047 | 7.24 ± 0.039 |
|  | pCO_2_ (*μ*atm) | 451 ± 7.30 | 456 ± 8.38 | 2977 ± 157 | 2777 ± 77.9 |
|  | Ω_calcite_ | 2.77 ± 0.05 | 2.65 ± 0.14 | 0.53 ± 0.02 | 0.56 ± 0.07 |
|  | Total DIC (*μ*mol L^-1^) | 1676 ± 21.8 | 1648 ± 55.4 | 1867 ± 28.4 | 1842 ± 113 |
|  | CO_3_^2-^ (*μ*mol L^-1^) | 111 ± 2.05 | 106 ± 5.64 | 21.4 ± 0.959 | 22.5 ± 2.71 |
|  | Alkalinity (TA) | 1834 ± 23.6 | 1799 ± 61.8 | 1806 ± 25.6 | 1789 ± 116 |
|  | Salinity | 29.1 ± 0.4 | 29.1 ± 0.4 | 29.1 ± 0.4 | 29.1 ± 0.4 |
|  | Temperature (°C) | 24 ± 2.2 | 24 ± 2.2 | 24 ± 2.2 | 24 ± 2.2 |
|  | Dissolved oxygen (μM) | 222 ± 13.6 | 126 ± 23.9 | 223 ± 13.7 | 117 ± 8.45 |
| Experiment 3 | pH_T_ | 7.94 ± 0.012 | 7.94 ± 0.010 | 7.33 ± 0.040 | 7.20 ± 0.031 |
|  | pCO_2_ (*μ*atm) | 508 ± 31.4 | 524 ± 5.34 | 2414 ± 324 | 2997 ± 140 |
|  | Ω_calcite_ | 3.33 ± 0.01 | 3.20 ± 0.13 | 0.93 ± 0.17 | 0.68 ± 0.01 |
|  | Total DIC (*μ*mol L^-1^) | 1815 ± 49.5 | 1805 ± 49.5 | 2015 ± 49.5 | 1943 ± 60.1 |
|  | CO_3_^2-^ (*μ*mol L^-1^) | 132 ± 0.41 | 127 ± 5.42 | 36.8 ± 6.92 | 26.9 ± 0.38 |
|  | Alkalinity (TA) | 1998 ± 46.4 | 1981 ± 55.6 | 2000 ± 68.6 | 1897 ± 56.2 |
|  | Salinity | 29.3 ± 1 | 29.3 ± 1 | 29.3 ± 1 | 29.3 ± 1 |
|  | Temperature (°C) | 24 ± 1 | 24 ± 1 | 24 ± 1 | 24 ± 1 |
|  | Dissolved oxygen (μM) | 269 ± 7.22 | 124 ± 22.3 | 269 ± 8.15 | 127 ± 15.8 |
