# Supplementary material for: Individual and combined effects of low dissolved oxygen and low pH on survival of early stage larval blue crabs, Callinectes sapidus
Source: PLoS One. 2018 Dec 7;13(12):e0208629. doi: 10.1371/journal.pone.0208629 (PMC6285982; doi:10.1371/journal.pone.0208629)
Supplement: S2 Table — (DOCX) [file pone.0208629.s002.docx]

**S2 Table**. **Mean pH, carbonate chemistry, alkalinity, dissolved oxygen, temperature and salinity (± 1 SD) during experiment four and five in which larval stage *Callinectes sapidus* were exposed to differing levels of pH and dissolved oxygen achieved via mixing tanked gases.**

| **Experiment** | **Parameter** | **Control** | **Low oxygen** | **Low pH** | **Low oxygen, low pH** |
| --- | --- | --- | --- | --- | --- |
| Experiment 4 | pH_T_ | 7.92 ± 0.03 | 7.94 ± 0.02 | 7.39 ± 0.05 | 7.32 ± 0.01 |
|  | pCO_2_ (*μ*atm) | 515 ± 48.5 | 480 ± 27.2 | 2012 ± 114 | 2295 ± 28.2 |
|  | Ω_calcite_ | 3.00 ± 0.20 | 3.14 ± 0.23 | 1.01 ± 0.07 | 0.90 ± 0.02 |
|  | Total DIC (*μ*mol L^-1^) | 1730 ± 14.1 | 1715 ± 21.2 | 1930 ± 14.1 | 1945 ± 7.07 |
|  | CO_3_^2-^ (*μ*mol L^-1^) | 119 ± 8.13 | 125 ± 9.13 | 40.2 ± 2.92 | 35.7 ± 0.72 |
|  | Alkalinity (TA) | 1897 ± 0.31 | 1892 ± 34.7 | 1933 ± 21.6 | 1933 ± 8.89 |
|  | Salinity | 29.3 ± 1 | 29.3 ± 1 | 29.3 ± 1 | 29.3 ± 1 |
|  | Temperature (°C) | 24 ± 1 | 24 ± 1 | 24 ± 1 | 24 ± 1 |
|  | Dissolved oxygen (μM) | 267 ± 4.39 | 68.7 ± 19.5 | 273 ± 2.36 | 76.2 ± 14.1 |
| Experiment 5 | pH_T_ | 7.97 ± 0.02 | 7.96 ± 0.01 | 7.34 ± 0.06 | 7.29 ± 0.06 |
|  | pCO_2_ (*μ*atm) | 484 ± 42.0 | 488 ± 20.0 | 2096 ± 2.81 | 2563 ± 243.3 |
|  | Ω_calcite_ | 3.55 ± 0.03 | 3.42 ± 0.02 | 1.08 ± 0.04 | 0.86 ± 0.13 |
|  | Total DIC (*μ*mol L^-1^) | 1835 ± 63.6 | 1810 ± 42.4 | 2035 ± 35.4 | 2005 ± 63.6 |
|  | CO_3_^2-^ (*μ*mol L^-1^) | 141 ± 1.51 | 136 ± 1.13 | 42.9 ± 1.44 | 34.1 ± 5.56 |
|  | Alkalinity (TA) | 2031 ± 58.1 | 1999 ± 42.1 | 2038 ± 37.0 | 1982 ± 78.4 |
|  | Salinity | 29.3 ± 1 | 29.3 ± 1 | 29.3 ± 1 | 29.3 ± 1 |
|  | Temperature (°C) | 24 ± 1 | 24 ± 1 | 24 ± 1 | 24 ± 1 |
|  | Dissolved oxygen (μM) | 261 ± 7.07 | 77.3 ± 9.50 | 266 ± 9.06 | 83.0 ± 6.41 |
