# Supplementary material for: Individual and combined effects of low dissolved oxygen and low pH on survival of early stage larval blue crabs, Callinectes sapidus
Source: PLoS One. 2018 Dec 7;13(12):e0208629. doi: 10.1371/journal.pone.0208629 (PMC6285982; doi:10.1371/journal.pone.0208629)
Supplement: S3 Table — (DOCX) [file pone.0208629.s003.docx]

**S3 Table**. **Experiments one, two, and three: two-way analysis of variance for *Callinectes sapidus* larval survival when exposed to two levels of dissolved oxygen and pH for 14 days.**

| ***Experiment*** | ***Source of variation*** | ***df*** | ***SS*** | ***MS*** | ***F-value*** | ***p-value*** |
| --- | --- | --- | --- | --- | --- | --- |
| Experiment 1 | Dissolved oxygen | 1 | 0.06332 | 0.06332 | 7.475 | 0.01813 |
|  | pH | 1 | 0.12615 | 0.12615 | 14.891 | 0.00227 |
|  | Dissolved oxygen and pH | 1 | 0.03203 | 0.03203 | 3.781 | 0.07564 |
|  | Residuals | 12 | 0.10166 | 0.00847 |  |  |
|  | Total | 15 | 0.32316 | 0.22997 |  |  |
| Experiment 2 | Dissolved oxygen | 1 | 0.4907 | 0.4907 | 27.749 | 0.00020 |
|  | pH | 1 | 0.1637 | 0.1637 | 9.259 | 0.01219 |
|  | Dissolved oxygen and pH | 1 | 0.0001 | 0.0001 | 0.007 | 0.93286 |
|  | Residuals | 12 | 0.2122 | 0.0177 |  |  |
|  | Total | 15 | 0.8667 | 0.6722 |  |  |
| Experiment 3 | Dissolved oxygen | 1 | 0.15707 | 0.15707 | 17.539 | 0.00126 |
|  | pH | 1 | 0.09589 | 0.09589 | 10.708 | 0.00667 |
|  | Dissolved oxygen and pH | 1 | 0.04138 | 0.04138 | 4.621 | 0.05267 |
|  | Residuals | 12 | 0.10747 | 0.00896 |  |  |
|  | Total | 15 | 0.40181 | 0.30330 |  |  |
