# Supplementary material for: Individual and combined effects of low dissolved oxygen and low pH on survival of early stage larval blue crabs, Callinectes sapidus
Source: PLoS One. 2018 Dec 7;13(12):e0208629. doi: 10.1371/journal.pone.0208629 (PMC6285982; doi:10.1371/journal.pone.0208629)
Supplement: S4 Table — (DOCX) [file pone.0208629.s004.docx]

**S4 Table. Experiments four and five: two-way analysis of variance for *Callinectes sapidus* larval survival when exposed to two levels of dissolved oxygen and pH for 4 days.**

| **Experiment** | **Source of variation** | ***df*** | ***SS*** | ***MS*** | ***F-ratio*** | ***p-value*** |
| --- | --- | --- | --- | --- | --- | --- |
| *Experiment 4 | Dissolved oxygen | 1 | 5.183 | 5.183 | 595.1 | <0.00001 |
|  | pH | 1 | 0 | 0 | 0 | 0.996 |
|  | Dissolved oxygen and pH | 1 | 0 | 0 | 0 | 0.996 |
|  | Residuals | 12 | 0.105 | 0.009 |  |  |
|  | Total | 15 | 5.288 | 5.192 |  |  |
| Experiment 5 | Dissolved oxygen | 1 | 4.172 | 4.172 | 204.014 | <0.00001 |
|  | pH | 1 | 0.011 | 0.011 | 0.523 | 0.483 |
|  | Dissolved oxygen and pH | 1 | 0.016 | 0.016 | 0.795 | 0.390 |
|  | Residuals | 12 | 0.245 | 0.020 |  |  |
|  | Total | 15 | 4.444 | 4.219 |  |  |

*Experiment 4 violated the assumption of homogeneity of variance (due to 100% mortality, and thus lack of variance among replicates within the low DO treatments).
