# Supplementary material for: Individual and combined effects of low dissolved oxygen and low pH on survival of early stage larval blue crabs, Callinectes sapidus
Source: PLoS One. 2018 Dec 7;13(12):e0208629. doi: 10.1371/journal.pone.0208629 (PMC6285982; doi:10.1371/journal.pone.0208629)
Supplement: S5 Table — (DOCX) [file pone.0208629.s005.docx]

**S5 Table**. **Logistic, 3-parameter nonlinear regression for *Callinectes sapidus* larval survival when exposed to varying levels of dissolved oxygen for a period < 4 days; Regression Equation: y = a / [1 +(x/x_0_)^b^]**

| **R** | **Rsqr** | **Adj. Rsqr** | **SE of estimate** |
| --- | --- | --- | --- |
| 0.9060 | 0.8209 | 0.8150 | 13.2772 |

| **Parameter** | **Coefficient** | **SE** | **t** | **P** |
| --- | --- | --- | --- | --- |
| a | 69.5614 | 2.3529 | 29.5639 | <0.0001 |
| b | -9.2293 | 2.3115 | -3.9927 | 0.0002 |
| X_0_ | 108.9538 | 3.2301 | 33.7304 | <0.0001 |
