# Supplementary material for: Individual and combined effects of low dissolved oxygen and low pH on survival of early stage larval blue crabs, Callinectes sapidus
Source: PLoS One. 2018 Dec 7;13(12):e0208629. doi: 10.1371/journal.pone.0208629 (PMC6285982; doi:10.1371/journal.pone.0208629)
Supplement: S6 Table — (DOCX) [file pone.0208629.s006.docx]

**S6 Table**. **Linear regression for *Callinectes sapidus* larval survival when exposed to varying dissolved oxygen levels for 14 days; Regression Equation: y = 0.109x – 4.554**

| **R** | **Rsqr** | **Adj. Rsqr** | **SE of estimate** |
| --- | --- | --- | --- |
| 0.511 | 0.261 | 0.245 | 11.035 |

| **Parameter** | **Coefficient** | **SE** | **t** | **P** |
| --- | --- | --- | --- | --- |
| Y-Intercept | -4.554 | 5.080 | -0.896 | 0.375 |
| Slope | 0.109 | 0.0271 | 4.031 | <0.001 |
