# Supplementary material for: Individual and combined effects of low dissolved oxygen and low pH on survival of early stage larval blue crabs, Callinectes sapidus
Source: PLoS One. 2018 Dec 7;13(12):e0208629. doi: 10.1371/journal.pone.0208629 (PMC6285982; doi:10.1371/journal.pone.0208629)
Supplement: S7 Table — (DOCX) [file pone.0208629.s007.docx]

**S7 Table**. **Linear regression for *Callinectes sapidus* larval survival when exposed to varying pH levels for 14 days; Regression Equation: y = 16.325x – 108.96.**

| **R** | **Rsqr** | **Adj. Rsqr** | **SE of estimate** |
| --- | --- | --- | --- |
| 0.426 | 0.182 | 0.164 | 11.614 |

| **Parameter** | **Coefficient** | **SE** | **t** | **P** |
| --- | --- | --- | --- | --- |
| Y-Intercept | -108.96 | 38.816 | -2.807 | 0.007 |
| Slope | 16.325 | 5.111 | 3.194 | 0.003 |
